# A Qualitative Exploration of the Lived Experiences and Perspectives of Equine-Assisted Services Practitioners in the UK and Ireland

**DOI:** 10.3390/ani15152240

**Published:** 2025-07-30

**Authors:** Rita Seery, Lisa Graham-Wisener, Deborah L. Wells

**Affiliations:** Animal Behaviour Centre, School of Psychology, Queen’s University Belfast, Belfast BT7 1NN, UK; l.graham-wisener@qub.ac.uk (L.G.-W.); d.wells@qub.ac.uk (D.L.W.)

**Keywords:** Equine-Assisted Services, equine and human wellbeing, horses, one health, practitioners, qualitative research, reflexive thematic analysis

## Abstract

**Simple Summary:**

Equine-Assisted Services (EAS) include horses as part of a treatment, learning, or therapeutic strategy with the view to improving human wellbeing. Until now, the perspectives of those who provide these services have received little attention. To address this gap, 15 EAS practitioners from the UK and Ireland were interviewed regarding their views on the horse–human bond, how horses affect change in clients, as well as challenges inherent in this field. Five themes were uncovered from the data. Practitioners believe that horses provide key benefits in regard to relationship and relational skills development, as well as supporting client motivation to engage in a wide range of different services. However, concerns were raised with regard to practitioner competencies to practice, training provisions, and governance. Further work is required to ensure optimal efficacy, safety and standards are being attained within EAS.

**Abstract:**

Equine-Assisted Services (EAS), which incorporate horses in a variety of ways in an effort to improve human wellbeing, have grown in popularity in recent years. Although much research has been conducted regarding the benefits that horses may provide for human health and wellbeing, little attention has been paid to practitioners’ experiences and perspectives of the field, despite the fact they are uniquely positioned to advance our understanding of this area. This study aimed to explore practitioners’ lived experiences of EAS, focusing on the benefits they observed, possible underlying mechanisms for any health benefits witnessed, and challenges faced in the area. Fifteen EAS practitioners from the UK/Ireland took part in qualitative semi-structured interviews, analysed using reflexive thematic analysis. Five themes were identified, three of which related to the horse’s influence on building connections, relationships, and enriching the process, whilst the remainder explored challenges within the field of EAS. These themes were explored through the practitioners’ lens, where possible linking them to our current understanding of human–animal interactions and related fields in the literature. Findings showed that horses, through EAS, were considered invaluable for building relationships, relational skills, and motivation to engage in whichever service was being provided. However, EAS was also viewed as complex. Concerns regarding competencies to practice, training, and lack of governance were expressed. These areas need further exploration and progress if EAS is to grow in efficacy and attain professional status.

## 1. Introduction

Equine-Assisted Services (EAS) is an umbrella term used to describe a variety of services that incorporate horses with the intent of improving human wellbeing. These services may be mounted or ground-based, often with an emphasis on building relationships, particularly between horse and client.

Much evidence has been accrued to support the health advantages of horses for a wide range of cohorts, including individuals with Autism Spectrum Disorder (ASD) [1,2,3], Attention Deficit Hyperactivity Disorder (ADHD) [4], Post Traumatic Stress Disorder (PTSD) [5], depression [6], anxiety [7], and a range of other conditions [8]. In addition, purposely directed mounted or riding activities have been shown to be beneficial to the physical and/or emotional health of cohorts affected by, amongst other conditions, cerebral palsy [9], multiple sclerosis [10], and speech delay [11].

Although there is a growing body of evidence which supports the benefits of horses for human health and wellbeing, the underlying mechanisms at play have, to date, proved elusive to establish. Gaining insights into the experiences of the clients themselves is an obvious avenue to explore to this end, and indeed a number of qualitative studies have focused on the lived experiences of clients and their caregivers [12,13,14,15]. Fewer studies, however, have explored the experiences of EAS practitioners, a population that witness firsthand the dynamics of client-horse interactions and unfolding progress in a real-time natural setting. Practitioners are uniquely positioned to cast their views on the perceived characteristics of effective practice, identify possible concerns, and offer considered solutions to the field going forwards.

Recent quantitative research has brought to light some of the previously undocumented practice patterns, experiences, and perspectives of practitioners across the EAS spectrum [16]. This study confirmed common anecdotal reports and shed useful light on the more recurrent themes and outcomes experienced through EAS from the perspective of practitioners. The findings pointed to the multifaceted nature of EAS and highlighted some broad areas of concern, including training, knowledge, welfare, and a need to professionalise the sector. These general findings are in line with what might be expected from a survey-based quantitative study, allowing a macro-level view of the area under investigation [17].

While quantitative research can provide us with numerical evidence to support a hypothesis, it often fails to convey the real-life embodied experience of individuals, leading to more one-dimensional findings. Qualitative research, by contrast, draws on multiple perspectives, with the view that gaining a greater understanding of a phenomenon requires looking at it from a variety of angles [18]. This necessitates an open mind and a level of awareness and knowledge regarding the context in which the phenomenon is situated [19]. A qualitative approach can generally probe deeper into the complexities of human experience to reveal different forms of knowledge [18]. In addition, qualitative investigations can give voice to multiple perspectives, allowing these to be communicated and shared more widely [20]. In particular, this method can capture the beliefs, values, and attitudes that may be missed in more quantitative research [21].

To date, qualitative research in the field of EAS has focused on clients, guardians and very specific practitioner groups or models [11,12,13,14,15]. The following study therefore aimed to investigate the wider field of EAS and horse–human bond through the perspectives of EAS practitioners using a qualitative methodology. More specifically, it sought to examine practitioners’ perspectives through the following research questions; (1) What is the experience and perception of EAS practitioners in terms of the impact of the horse–human bond for wellbeing? (2) What do practitioners consider to be the key underlying mechanism/s of action of the EAS they deliver? (3) What do practitioners feel may contribute to, or impede, the delivery of the EAS they provide? It was hoped that the investigation would further add to the existing knowledge in this area, thus far collected using quantitative approaches, contributing greater depth and diversity of knowledge capable of facilitating beneficial changes at both the micro-practitioner and macro-policy level.

## 2. Materials and Methods

### 2.1. Study Design and Approach

Cross sectional, semi-structured interviews were carried out to explore practitioners’ perspectives of EAS. A reflexive thematic analysis was employed for this study. This is a method of inquiry that allows the researcher to explore the subjective experiences of participants and, through their reflections and thoughts, gain insights into a phenomenon and identify possible mechanisms of change [22]. The approach is flexible and not tied to any philosophical or epistemological assumptions, allowing a critical realist approach to be implemented. Reflexive thematic analysis put forward by Braun and Clarke (2021) was adopted [23], whilst also following recent reflexive thematic analysis reporting guidelines [24]. Here, the researcher takes an active part in the generation of knowledge, with researcher subjectivity being an important part of the process. Researcher experiences and perspectives, together with those of participants, are viewed as enriching the narratives and interpretation within a reflexive thematic analysis paradigm. However, researcher reflexivity throughout the research process is seen as essential in ensuring the participants’ voice is fairly represented.

A philosophical lens of critical realism was employed. Ontologically, critical realism is situated between a realist and relativist standpoint. This position proposes that reality may only be partially observed, since human knowledge can only ever encompass part of a vast reality [25]. Epistemologically, critical realism recognises that our understanding of reality depends on individual perspectives, including the context or resources present. From an epistemological stance, critical realism can be seen as a particular blend of realism and constructivism, accepting that reality can be partially observed empirically, while also being moderated by social and other contexts [26]. Critical realism is a useful philosophical foundation for exploring practitioner perspectives within EAS, allowing a deeper understanding of the underlying causal processes which cannot be directly observed. This aligns well with a systems thinking approach, highlighting the interconnectedness inherent within complex systems, such as those found within EAS. As this field comprises a rich social and relational component, a full understanding through quantitative means is difficult to achieve. However, through reflexive thematic analysis and critical realist positioning, one can explore causality, effect, and underlying mechanisms in multiple ways while recognising social processes and perspectives which form a natural alignment with the goals of reflexive thematic analysis.

### 2.2. Researcher Reflexivity

The interviewer (RS) was a PhD student with a background in Equine Science. As part of the reflexive thematic analysis conducted, RS acknowledged that her background, personal experiences, beliefs, and values played a significant role in shaping this research, with horses being a very important part of her life since early childhood. Having worked, or taken part, in a myriad of equine activities, from pony club, low-level competition, breeding, and teaching riding and horse management, RS has been exposed to many different types of horses, people working with horses, training methods, management practices, attitudes, and also motivations for associating with horses. Early experiences with horses as a young child represented an opportunity to bond with a non-human being. The traditional riding school environment introduced RS to riding and finding a community of like-minded people. However, more recently, the motivation to be with horses has become more relational. The knowledge and experience of RS has led her to being well-placed to understand the perspectives of ‘horse people’. In addition, a limited knowledge of EAS through, for a short time, providing equine-assisted learning sessions, as well as volunteering for riding for the disabled groups, have been highly relevant for this study. In this way, an insider-outsider position could be said to be held. RS has worked hard to acknowledge and reflect on her personal situation and biases, maintaining a reflexive diary before and throughout the interviews, as well as interrogating all codes, themes, and interpretations throughout.

### 2.3. Participant Recruitment

Participants were purposively sampled to include practitioners spanning a range of backgrounds and EAS categories, e.g., equine-assisted physical therapy, mental health therapy, learning, therapeutic riding, or horsemanship. Those recruited had either expressed an interest in taking part in research through previous contact with the researcher (from a previous quantitative survey, [16]) or as a result of answering an open call for participants put forward via social media, print media, or word of mouth. A very small number of participants were invited directly via snowballing to ensure a purposeful sample was achieved. Most of the interviewees were either not known to the researcher before the recruitment stage or had become acquainted through the previous study. A small number had an earlier acquaintance with the researcher through a mutual interest in horses. Only practitioners who were over 18 years of age, from the UK or Ireland, and who currently or previously offered EAS (>8 sessions/month) for at least 12 months, were eligible to participate. While it was not a requirement that participants were a member of an EAS register (HEIR or Athena Herd Foundation), participants were screened to ensure basic eligibility for registration (e.g., EAS qualification, extensive experience, service-related qualification). All participants, except for one retired practitioner, were involved in EAS work at the time of interview. 16 participants were recruited with one participant withdrawing before interview stage (Participant 9). A sample size of 15 was deemed acceptable due to the exploratory nature of the study, the diversity of participant backgrounds and services, and the rich and detailed accounts that were subsequently attained. These elements, in addition to the researchers’ immersion and reflexivity regarding the data, ensured the subsequent greater information power contained within sample required fewer overall participants [27,28].

All potential participants were e-mailed a Participant Information Sheet and sample consent form and given an opportunity to ask questions. Following this, participants were invited to complete a brief screening survey to collect some basic practitioner information and to ensure all study inclusion criteria were met, along with a consent form to be completed electronically. Once screening was concluded and consent obtained, participants were invited to partake in the interview at a time and location convenient to both parties.

### 2.4. Data Collection and Preparation

A semi-structured interview format was utilised for this study. All interviews, which took place between December 2022 and July 2023, were conducted by the lead author (RS), with verbal consent re-confirmed before commencement. Interviews were audio-recorded and took place either at the participants’ home/workplace or remotely via Zoom.

Interviews were conducted with the aid of an interview guide developed specifically for this study. The guide consisted of questions related to the practitioners’ own experience of the horse–human bond (e.g., ‘What does the horse–human bond mean to you?’), the service provided (e.g., ‘Can you explain to me what you do in terms of EAS work?’), practitioners’ understanding and experience of what is happening during and after sessions (e.g., ‘What impacts have you found for clients?’,) and possible mechanisms of change (‘What do you think is happening within the horse–human interaction that contributes to this change or impact?’). The latter part of the interview explored the challenges practitioners (or the EAS field) face in providing these types of services and possible solutions moving forward (‘Have you experienced any challenges or have any concerns in relation to your own practice or for the Equine Assisted field in general?’, Are there any changes you would like to see?).

A single interview was conducted with each participant, lasting an average of 60 min (range ~45–90 min). All interviews were transcribed with the aid of basic transcription software (Microsoft built in transcribe feature) followed by careful manual transcription in preparation for thematic analysis to follow.

An inductive approach was used to code the data, using a combination of initially semantic, but later latent, code generating styles. A bottom-up approach was taken, whereby code generation led to the organic development of initial themes without any prior influence of theory, following a reflexive thematic analysis ethos. Theme names were developed which best conveyed the shared meaning expressed by the participants.

### 2.5. Maintaining Research Transparency

An audit trail was maintained to promote transparency; this involved, documenting all stages of the research process, including data collection, coding, theme development and interpretation. Mind maps and other visual techniques were used to develop themes and connections in a coherent and progressive way. Reflexive journaling was employed throughout data collection and the analysis process to anchor the idea generation and raise awareness of areas which may have been overlooked due to researchers’ previous assumptions.

### 2.6. Reflexive Thematic Analysis

Data generated were analysed by RS following Braun and Clarke’s (2006) six phases of reflexive thematic analysis [29]:

#### 2.6.1. Phase 1: Familiarisation

This stage requires a deep immersion into the data to get to know the depth and breadth of perspectives contained within the transcripts. The transcripts were read several times, while the audio recordings were listened to on multiple occasions in different settings (at home, in the car, before and after horse observations) to facilitate the generation of new connections and ideas from the dataset.

#### 2.6.2. Phase 2: Generating Initial Codes

Once it was felt that adequate familiarisation had been achieved, coding of the data commenced. Complete coding was undertaken, i.e., going through the entire dataset to code all relevant data. Most codes consisted of brief phrases. Coding took place by hand using sticky notes, with codes assigned interview and line numbers to refer back to later. Relevant data extracts were also highlighted at this stage. Coding was initially semantic, or data-driven, especially during the first-level coding stage where large numbers of codes were generated. During the second stage of coding, very similar codes were merged and re-named to reflect the shared meaning of the codes. With a deeper understanding of the data, more latent codes were generated. Following this, codes were collated into loose categories in preparation for theme development.

#### 2.6.3. Phase 3: Searching for Themes

Phase 3 involved gathering the codes together in a single area and assembling them into groups with similar meanings as a prequel to theme generation. This stage required multiple sittings to fully grasp what kind of story the data might be telling. Codes were grouped into various candidate themes, re-arranged, reworked, and reworded. Time was spent ‘trying out’ themes to see if they provided a coherent and true account of the data.

#### 2.6.4. Phase 4: Reviewing Themes

Following Phase 3, a thematic tree, consisting of candidate themes, subthemes, a sample of codes, and data extracts, was created. This phase was marked by further interrogation of the data, as the candidate themes were once again questioned and cross-examined. At the same time codes were revisited.

#### 2.6.5. Phase 5: Defining and Naming Themes

At this stage, naming and defining of themes was informed, similar to the earlier stages, through a critical realist lens. This was particularly beneficial due to the widespread use of metaphor among many EAS modalities.

### 2.7. Ethical Considerations

This study was given full ethical approval by the Faculty Research Ethics Committee, Queen’s University Belfast (EPS 22_356).

## 3. Results

### 3.1. Participant Demographics

Fifteen participants from across the UK and Ireland took part in this study (Table 1). All of the participants were female and aged between 30 and 70 years, with most individuals over the age of 50. The participants together covered all 4 EAS categories, although the majority focused, to some degree, on equine-assisted learning. Most practitioners (*n* = 12, 80%) provided services for children and adolescents, with a slightly smaller number (*n* = 9, 60%) catering for adults. Only 2 practitioners (13.3%) reported providing services for clients over 65 years of age. The vast majority of individuals (*n* = 14, 93.3%) reported having 10+ years of equine-related experience. In terms of EAS-specific experience, practitioners were relatively evenly split between those that had up to 5 years of experience (*n* = 4, 26.7%), 5 to 10 years (*n* = 6, 40%), and over 10 years (*n* = 5, 33.3%). All of the participants reported having received EAS-related training. Although a wide range of training providers were sourced by practitioners (28 in total), the most frequently cited organisations were the Equine Assisted Growth and Learning Association (EAGALA), Riding for the Disabled Association (RDA), and Festina Lente. Just one practitioner cited on-the-job training as a training type. Multiple trainings from more than one organisation was a common feature of practitioners’ EAS education (*n* = 8, 53%).

### 3.2. Themes

**Figure 1:**
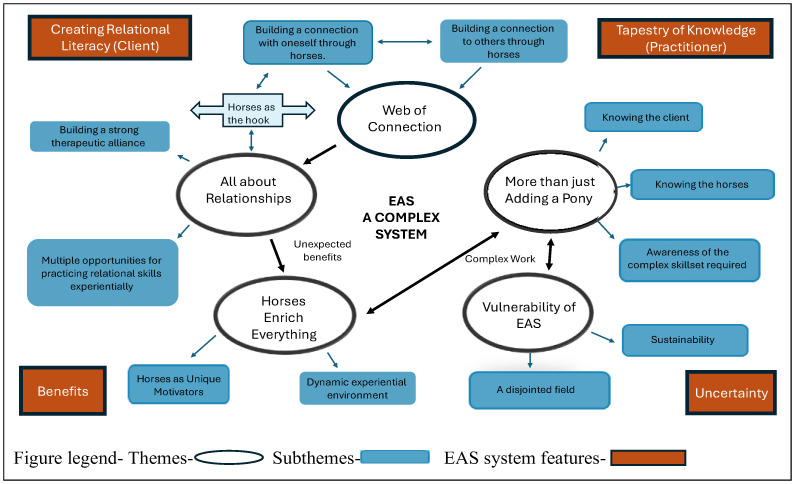
Developing the thematic web. The web includes five themes, candidate subthemes, and interconnections, including four key features associated with EAS.

Figure 1 illustrates the unfolding thematic development during the later stages of reflexive thematic analysis, including recognising EAS as a complex system with key features, such as uncertainty, unexpected benefits, non-linearity (expressed by practitioners), and a high degree of interconnectedness. Five interconnected themes evolved during the analysis. These were later refined into the themes and subthemes presented in Table 2.

#### 3.2.1. Theme 1. Developing Strong and Lasting Connections Through Horses

The most obvious theme to emerge from the data was that of connection. Connection was seen as the starting point for everything by many practitioners, with connection in all of its guises a frequent theme that wove its way through many of the transcripts. This connection was not limited to that experienced by clients. Having themselves experienced positive wellbeing through their connection to horses, the practitioners often felt well placed to share and appropriately promote such experiences to others. Having multiple opportunities to connect in a variety of ways (with horses, others, and/or the environment) was viewed as an inherent part of EAS.

##### Subtheme: Connection to Horses and the Self

Practitioners’ own connection to horses in many ways sets the scene for how practitioners understand the link between connection to horses and connection to the self, a kind of understanding by experiencing. Most practitioners provided vivid childhood memories describing the strength of connection they felt to horses, with many considering it a lifelong obsession. This connection with horses was often perceived as integral to the wider connection to oneself. For example, one participant indicated:

*‘the connection with horses has been the grounding…and that connection with horses has given me that place where I can just be…when everything else has, and there have been times, where everything else has just fallen away, and the horses have been a steadying point for me’*

(Participant 5)

Some described horses as their ‘first love’. A few practitioners came to work with horses later in their lives, but also spoke of the deep connection that they had with these animals, in some cases changing the trajectory of their lives. While practitioners often expressed this connection in terms of a direct horse–human experience, the connection was also described as being felt or experienced indirectly in multiple ways, with the horse as the catalyst. For example, practitioners cited horses as facilitating a greater connection to oneself, accepting themselves, being more grounded, or growing a sense of purpose. As one practitioner stated:

*‘There’s something about that relationship and its simplicity, in just being with the horse, just being yourself, them being themselves with you…that feeling…connecting with them and feeling and it’s also nice to feel like they are getting something back from you’*

(Participant 7)

Although practitioners often talked of their connection to horses having a profound effect on clients, as well as themselves as a person, others highlighted the more subtle personal impacts associated with being with horses:

*‘The main thing they [horses] do for me is keeping me grounded, you know, if I’m having a bad day and I’m feeling a bit down they bring me up, and if I’m on top of the world, I’m thinking I’m a great one, they’ll rebalance me, you know, and that’s both the ground [work] and the horse-riding’*

(Participant 16)

For others, finding a true connection with horses was a revelation in terms of personal wellbeing and growth:

*‘[the realisation of]…and that ability to connect with the animal and do things together, and that was kind of, that was a big moment. I think. So I think I remember getting a lot from that in terms of confidence, self-esteem and [through] that sort of connection’*

(Participant 7)

Connection to horses was often perceived as a step to a more generalised connection, one which happens naturally within EAS. Clients’ feelings of improved connection to oneself, as facilitated through horses, were often expressed as a kind of embodied sensory experience, a grounding, likened to mindfulness practice. The benefits of the transition from being ‘in your head’ to being ‘in your body’, and connecting on a being-to-being level, were very obvious for many practitioners. This grounding was considered to be essential for developing self-awareness and body awareness:

*‘So having that like, really being in your body and really being aware of what you’re doing, with your seat, and your hands and your leg. It’s really…it’s like yoga or dance or any of those types of things. It really brings them down into…into the moment, and that really helps to calm anxiety’*

(Participant 12)

Another practitioner stated:

*‘Sometimes we might have a little bit of an idea of what’s going on in our head but, just from doing the work, very few seem to know what’s going on in their bodies and their soul and the horses just join dots for people’*

(Participant 16)

This embodied connection facilitates a more experiential type of learning that seems to happen spontaneously. A ‘connection of understanding’, as described below, appears to be a cover mechanism at play, an understanding based on knowing the horse, attuning to where they are, and learning from, and through, this in an embodied way:

*‘…one of the things that horses teach is, you can be grounded, you can be calm…but even if a horse is startled and runs, at some point, they turn around and look back and stop, and it’s that sort of analogy of ‘It’s ok not to be ok’, but now we have to work out how we can get you back to being ok. So when you’re watching the horses you’re getting that connection of understanding’*

(Participant 3)

##### Subtheme: Connection to Others

For some, developing a connection with another being is the start of the journey of understanding and personal growth, but for others it can be a destination in itself. Lack of connection or a ‘connection deficit’ was implied by many as a very real phenomenon. Practitioners spoke of clients who initially were unable to connect with others due to mental or physical health issues, difficult childhood experiences, social exclusion or disadvantage, or being unable or unwilling to engage in a normal school or social environment to name but a few instances. Horses, being to some degree non-judgemental and fully present, were regarded as ideally suited to facilitating clients to be, and accept, themselves, allowing those first steps towards connection to others:

*‘[the horse] is giving that…is this sense of connection, belonging, grounding, mindfulness, less anxiety, less stressful. And it kind of supports the suggestion then that actually they’re not as useless as they thought they were, they’re not as bad as they thought they were, they’re not the label that people have given them. Because what the horse can do is see through all that shit…so let’s get [you] out to the field with the horses and let them, actually work out, what’s going on here’*

(Participant 3)

Connection in any shape or form can be a challenge for some. For those experiencing the greatest challenges, connecting with horses was considered by some to be a miracle in motion, a rare opportunity to connect, as outlined here:

*‘…and she’ll watch that pony eating, and she’s smiling and laughing, and, like she’s nearly moving her mouth to mimic what the pony’s doing. And it’s a level of connection that you wouldn’t get with a person. She wouldn’t give that attention to another person. She wouldn’t tune into their face, she wouldn’t be aware of their facial expression or movement of their face. So that [opportunity for] connection with something else is amazing’*

(Participant 7)

Connecting to horses was often viewed by practitioners as the ‘hook’ or ‘spark’ that starts a chain reaction, allowing for more broad connections to develop. Spontaneous conversation initiated by observing horses accelerates the finding of common ground and connection-building with the practitioner. This connection with horses, often highly valued by clients and easier to achieve than with humans, can act as the glue that keeps the client on track, sustaining the buy-in during more difficult times. The novel equine environment allows the client to experience new embodied sensations—new smells, natural or different surroundings, inclement weather, and other animals, in a naturalistic way. These new experiences, together with ‘being to being’ encounters, enable a web of connection that can situate clients. As one practitioner described it:

*‘…in the here and now, without the worry of the past or the fear of the future, and seeing other animals surviving perfectly well in that space’*

(Participant 11)

This ‘trinity of connection’ to oneself, nature, and others, encompasses the essence of what practitioners are aiming for as a base level for any EAS, serving as a prerequisite for the relationship building to follow.

#### 3.2.2. Theme 2. It’s All About the Relationships

Practitioners were keen to stress the pivotal and synergic influence that multiple- and multi-species relationships have on the efficacy of any EAS. The many opportunities to form relationships are considered not only a unique feature of EAS, but also its key strength, creating a milieu conducive to growth, while also building relational skills that can be readily transferred and used in the outside world.

##### Subtheme: Building a Strong Therapeutic Alliance

Although connecting to horses is perceived as the proverbial ‘hook’ that paves the way for engagement to follow, it is the subsequent development of quality relationships between the client and practitioner that are regarded as being fundamental to the success of EAS outcomes. Talking less, and taking time, were reported as common features of EAS sessions, particularly in the early stages, creating space for the clients to bond with the horses unhindered. Practitioners in this study frequently pointed to the important role that the horse plays in facilitating the rapid development of a practitioner-client relationship. In particular, many practitioners are very aware that clients judge their trustworthiness based on the type of practitioner-horse relationship on show, a kind of prototype for how they expect to be treated. One practitioner described the powerful influence the practitioner-horse relationship may have on clients’ perceptions:

*‘…but they probably would struggle [in mainstream therapy] because it’s kind of that traditional setting with the expectations. When we are working in a less traditional setting……they get to see how I am and judge how safe I am by how I interact with the horses. So they can see whether I am trustworthy, kind, supportive person with other beings before they [can] talk to me’*

(Participant 4)

This practitioner elaborated further by stating:

*‘So…that will give them a really good read on how I am going to treat them. If they see me being really pissed off with a horse or not very patient or talking to them in a certain way, than they have sussed out whether I am safe or not. So it’s really important how I embody the relationship in that moment…’*

(Participant 4)

Although practitioners often reported that clients could ‘suss them out’ so to speak, through their relationships with horses, the practitioner-horse relationship also served to model normal healthy relationships, allowing clients to see this in action. One practitioner described the practitioner-horse relationship as foundational:

*‘[without which] it’s hard then to facilitate a really high-quality positive session that positively impacts the client, [and] doesn’t negatively impact the horse by any means’*

(Participant 8)

A good practitioner-horse relationship also ensures that the horse’s wellbeing and viewpoint are considered, teaching empathy with others. Many practitioners felt that this demonstration of a good practitioner-horse relationship, along with positive client-horse experiences, helps develop a strong therapeutic relationship, as stated here:

*‘So when they have on the floor confidence as I like to call it…I give them little tasks to do, something simple…go rub the horses, groom, or whatever…to get the connection…and it could be taking the horse for a walk. And that then opens it up and it…It helps us build our therapeutic relationship massively as well because she’s after achieving to us something that was little but to her, it was the world, you know. And from that then I go into the deeper stuff’*

(Participant 16)

##### Subtheme: Multiple Opportunities to Practice Relationship Skills Experientially

Multiple relationships are a key feature of EAS, setting them apart from many other dyadic therapies or interventions. Having multiple parallel opportunities to develop better ‘relational literacy’—the ability to understand and practice healthy relations/relationships—something that many clients have trouble with, is perceived by practitioners as a strength of EAS. Participants spoke of the advantages of developing this relational literacy in real-time in a natural, non-clinical environment, through observation, direct interaction with horses, practitioners, and/or assistants, and improving their own relationship with themselves. The complex set of multiple and multispecies relationships at play, although not without risk, can have cumulative relational value:

*‘When you add more team players it makes the relationship more complex. So it’s the therapist, it’s the horse handler, it’s the side walker. So it’s the therapists’ relationship with each of these people, it’s their relationship with one another, and it’s the relationship with the client that can all benefit the session or derail it……so the therapeutic relationship also becomes more complex but also can be very beneficial’*

(Participant 6)

A feature of building relationships in an equine environment is that you can see in real-time how healthy relationships are formed, e.g., developing trust, the importance of listening to others, reciprocity, or responding to another’s actions or appropriately, etc. Horses observe and respond to humans’ body language in a relatively consistent way, meaning communication is often ‘cleaner’ and easier for clients to learn and understand. This demands a degree of focus that can help the clients be more present. Developing relational skills, such as self-awareness, observation, empathy, and reciprocal interactions, are all enhanced in this more straightforward relational space, as demonstrated here:

*‘Emotionally and mentally the kids just do seem to be very grounded and present around the horses. I do think a lot of that comes from the immediate feedback that horses offer…if they go up and run up to the horse and the horse is a bit like—‘okay’ [not happy with it]—they’re thinking, well, I’ll have to change my behaviour’*

(Participant 6)

Interactions within real relationships are ever-changing and practitioners frequently described the benefits of the ‘realness’ through horses as highly beneficial for clients’ experiential learning within a healthy relational context:

*‘It’s about mutuality as well, you know, it’s about sometimes I’m having an off day and the horse is having an off day as well, it’s about allowing that to happen…and then the clients see the relationship that you’ve got, you know through the animals, through the horses…then they give you trust. They know that…they can see you can have a relationship without control’*

(Participant 13)

This rich relational environment, where there are many opportunities to observe, develop, and practice building relationships is unique to EAS. The realness, consistency, and ’cleaner’ communication, all play an important part in developing skills that can be transferred, as conveyed by this practitioner:

*‘…the consistency is a big thing for me because the horses aren’t as variable as people’*

(Participant 7)

Practicing skills in this safe, consistent environment can serve as a key step towards transferring these skills to navigate the real-world school, work, or home environment. Once quality relationships have been established outside of the horses themselves, the human relationships often gain greater traction:

*‘So he would be saying [at the start] he was really attached to the horses and really wanted to be with them…now when the horses come through he’s not interested, not that he’s not interested in the horses, but because he’s got a relationship with the other therapist and I and he’s missing that human contact. And he trusts us so he doesn’t have to hide behind the horses…so that’s just by osmosis’*

(Participant 13)

Observing, learning, taking part, building relationships, and then transferring and using the skills gained beyond the realm of EAS is the ultimate aim of most practitioners. The unique relational dynamics are seen as key in making this happen.

#### 3.2.3. Theme 3. ‘I Couldn’t Do This Without the Horses.’ Horses Enrich the Service and Clients’ Everyday Lives

Practitioners universally reported upon the value that horses add to whatever service is being delivered. The sentiment ‘I couldn’t do this without the horses’ was frequently expressed, implied, or paraphrased across EAS groups. Horses were seen to provide enrichment through their presence, the focus they offer, enjoyment, and the novelty aspects of experiential learning. Other less visible benefits, such as improved sleep, self-regulation, or family cohesion, were widely acknowledged as part and parcel of the unexpected ways that horses may indirectly enhance lives.

##### Subtheme: Horses and the EAS Environment as Unique Motivators

Although practitioners had various opinions as to why horses were so effective for what they wanted to achieve, there was also an air of not quite knowing why, or how, progress is made at times. Some practitioners tried to make sense of this uncertainty by pointing to the horse’s physiology as being influential:

*‘But there is something [about horses]. I don’t know if it’s because they’ve got such big hearts, such a big circulatory system going on. That it has, it does have some sort of influence on people. Whether it’s calming, whether it’s from magnetic fields, there is something real in that, but I don’t know what it is. But the effect is…is visible’*

(Participant 14)

The contrast between services that incorporate horses to more clinical or traditional interventions was frequently noted. Most practitioners perceived beneficial changes in the emotional state of clients within a short time of being in the presence of a horse. This ‘regulation’ or calming effect was seen as one of the main benefits of incorporating horses into specific services, helping clients to become more present and open to new learning. This was noted by both ground-based and mounted service providers, although the following statement specifically describes therapeutic riding:

*‘The thing that I have found…is that the movement provides such a therapeutic and regulatory effect. So that’s something that’s fantastic. They can come in and can be really heightened…and it’s really the movement of the horse that provides that relaxation’*

(Participant 6)

Many practitioners defined the initial period with the horse as enabling clients time to get into the zone where learning or dialogue could happen. Horses were also described as having a unique ability to grab the client’s attention, motivate engagement with the services, and create a milieu for growth:

*‘…lots of kids are…just more engaged because it’s a horse. If you’re more engaged you’re going to get more from it, you’re going to pay better attention, you’re going to be more connected to whatever it is you are working on, you’ll probably remember it more as well, because that’s, you know, a more memorable situation than sitting in a room with somebody’*

(Participant 7)

The fact that EAS is so unlike regular therapy or interventions seems to add to the appeal for clients, and practitioners were very much aware of this. Services that incorporate horses for wellbeing often have elements of fun and enjoyment that may not be typical of a traditional educational or therapy setting. This novel setting was often perceived as creating a shift in clients’ attitudes for the better:

*‘…when kids are going through or being referred for one-to-one counselling, it’s still very much an environment like school, which they’re not very keen about…which they rail against. Whereas if you bring them out into nature, and that you’re around the horses, and you know, there’s probably dogs and cats and chickens as well, to be honest. So it’s a completely different environment for them…because they know they will be working with these animals they seem to miraculously engage’*

(Participant 3)

##### Subtheme: Dynamic Experiential Environment Promotes Growth

EAS is dynamic in nature and demands flexibility on the part of the practitioner and client alike. Clients have to deal with change regularly, for example, getting ready to mount, moving from a stable environment to an arena or field, and learning how to interact effectively or sympathetically with horses. Coping and leaning into these inter-situational challenges in a supportive environment is regarded by practitioners as helping to grow confidence in dealing with change outside the realms of EAS:

*‘And the biggest change I find is that confidence growing through experiential learning and exposure to the horses and most people who come here terrified actually want to bring the horses home with them [after]. So it’s about how we embrace fear and how we embrace change’*

(Participant 2)

The largely experiential nature of equine-assisted services was also seen as hugely positive by many practitioners. Learning through observation and practice typified by many types of EAS was viewed by practitioners as adding an extra dimension for clients, allowing them to play a central role in their understanding and growth:

*‘It’s in front of them, they’re seeing, they’re nearly seeing their thoughts out in front of them, you know. And then it’s live, it’s organic because the horses are free, they can do what they want, no one’s prescribing it…it just makes it so real for them…I couldn’t achieve it in the office, yeah, I couldn’t achieve it in the office and I love my [normal] psychotherapy work’*

(Participant 16)

##### Subtheme: Unexpected Co-Benefits to Everyday Life

An unexpected perceived benefit of taking part in EAS was the positive everyday relational outcomes and how the clients were viewed outside the service. Transferable relational skills learned through EAS most likely impacted this shift. However, there was also a sense that clients might be viewed differently, or as more ‘normal’. Taking part in a service that includes horses or riding may help create a sense of pride or achievement not typical of other interventions, as described here:

*‘One of the areas we saw was around framing how children were spoken about. And one woman said—my mother always used to say ‘My daughter has a child with disabilities’ and now says ‘My granddaughter goes horse-riding’—and that for me, those are bigger steps than that big dramatic thing where a child learns to speak. Probably that was going to come at some stage…but those [family] connections are more important’*

(Participant 5)

In addition, taking part in an EAS could have positive impacts for families outside the relational benefits. Practitioners described profound changes in this respect, including improvements in school attendance and attitude, a more harmonious family life, or even allowing families to experience a more normal life through positive changes. These could be economical in nature. For example, a child who returns to regular schooling can free up a parent to return to some paid employment. In some cases, EAS can provide significant quality-of-life improvements that are hard to quantify:

*‘But what I hear all over is that the client sleeps better, not just on the days they had a session…but in general they started sleeping better. For a family where a Mom has to stand up to turn the child 14 times a night, then just being able to do that 9 times a night makes a huge difference…or lessening the amount of oxygen that you give the child…and that is the kinds of things we see all the time’*

(Participant 6)

#### 3.2.4. Theme 4. EAS Is More than Just Adding a Pony

A common thread in practitioners’ narratives was the consensus that this work was more than just adding a horse into the mix. Practitioners stressed the many skills and competencies needed to provide safe and effective service as well as the need for a deep understanding of clients and horses to ensure meaningful and respectful interactions.

##### Subtheme: Awareness Needed of the Strong and Complex Skillset Requirement

Practitioners described a complex, dynamic system requiring extensive horse, client, and service knowledge, interwoven with keen observation skills. The latter were regarded as crucial for the success and safety of clients, practitioners, and horses alike. Many practitioners mentioned the need for ‘engrained’ or ‘embodied’ knowledge, a kind of deep fluency in reading what was happening simultaneously for, and between, horse and human; this was viewed as closely linked to practitioners’ skills, experience, and knowledge. Flexibility, or an ability to ‘think on your feet’ to help engage clients and horses, or pre-empt problems, was frequently mentioned. An attitude of universal empathy was seen as important for distinguishing EAS from more one-sided relationships with horses in a sport or leisure context. However, these skills were not regarded as being widespread. Most practitioners acknowledged that there was a core of competent and capable people involved in EAS. However, many practitioners spoke of a lack of understanding and knowledge among those starting out, and, more worryingly, with those who had been in the field for a time. The phrase ‘conscious incompetence’ was mentioned by a few practitioners, with several also expressing concerns about the flippant, casual, or naive attitudes held by some equine professionals or horse owners aiming to start an EAS programme, as alluded to below:

*‘So you’ll see things on…Facebook like ‘Oh I’m starting to work with kids aged 10. I’ve got no experience of kids. What kind of things will I do with them? You know, I want to say ‘Don’t do it, because you’re not qualified’ You know, it gets people a bad reputation’*

(Participant 13)

This perceived casual attitude, coupled with a lack of understanding of working within a scope of practice appropriate to qualifications and skills held led another practitioner to conclude:

*‘I think we don’t necessarily know why or how it works, there’s a lot of theoretical explanations…but I don’t think we fully know yet. And that in some ways undermines it. There are a lot of people who don’t take it seriously and think that it’s just playing with horses…that you don’t have to have any qualifications. You’ve got some horses, you want to help people. Let’s just have a go, which isn’t massively safe or ethical and you wouldn’t see in a lot of [similar] industries’*

(Participant 4)

Some practitioners spoke of the current trend to include retired racehorses or rescue horses in EAS sessions, a narrative that often emphasises horses and humans healing together and/or as an ideal second career. Providing a new life for vulnerable horses was seen as admirable and problematic in equal measure. On the one hand, practitioners spoke of the important contribution that rescue or retired horses can play when given time to adjust and heal from past physical or emotional issues. However, several practitioners stressed the considerable skill and time necessary to rehabilitate such animals, with some never attaining the emotional stability and trust required. The skills needed to integrate these horses were viewed as very far removed from the idea of ‘just adding a pony’. One practitioner described the dangers of not thinking through the dynamics of mixing potentially traumatised horses and people:

‘And there’s also this philosophy that if you’re working with traumatised [clients]…get rescue horses that are traumatised…they will heal each other. So that’s like…so I’ll say ‘If you were going for counselling, if you wanted counselling, you would go to a counsellor that was totally f***ed up would you?’ Well no. So how would you think that…so you’ve got a group of traumatised horses and you’ve got these kids coming from probably an EBD (Emotional and Behaviour Disorder) school…’

(Participant 13)

##### Subtheme: Knowing the Client

The vulnerable nature of clients accessing services was well recognised. Some practitioners stated or implied that EAS was often viewed as an intervention of last resort by clients or guardians, something to try when other avenues of treatment had failed. Many practitioners spoke of having clients with a range of challenges, with physical, mental, or emotional issues being common for those taking part in both mounted or unmounted activities. Furthermore, it was commonly felt that clients seeking help for one area of concern were more inclined to disclose deeper issues to the practitioner due to the powerful relational nature of the work. For this reason, understanding and being aware of an individual’s scope of practice, including when to refer or report to higher authorities, was seen as an essential skill in EAS, requiring appropriate training. Although the ease with which a good therapeutic alliance could be built was often portrayed as a blessing, it did come with added challenges should the practitioner not fully understand their scope of practice or capabilities:

*‘You have to be very well informed in terms of trauma, nervous system regulation, disability, and just come from a really good place. You have to want to do it in a way that you’re going to preserve the integrity of your client and your horse and you have to try and find that happy medium and trust your instinct and trust your gut with that as you go’*

(Participant 8)

There was also a felt lack of general understanding regarding the difference between providing a horse-riding experience in a riding school context and therapeutic riding sessions provided by a competent and qualified instructor with a specialised skill set:

*‘So you have people offering therapeutic riding because they are putting people with disabilities on a horse but they have no training in what they are doing’*

(Participant 10)

The presumption that adding a pony or putting a client on horses is inherently beneficial, requiring little additional training or regulation beyond that of riding school or even horse ownership, was perceived to be very concerning. These concerns were not restricted to horse owners or professionals trying to help people through horses, however. Human health professionals were also considered to underestimate the extent of knowledge and competencies required regarding equine and equine–human interactions:

*‘I’ve been approached to work with people that have done the equine therapy as an add-on but aren’t horse people, have no horse background and that concerns me…if you don’t have the awareness and the background and the knowledge to read a horses body language that worries me a bit because…I don’t know how you would negotiate that space safely independently….I’ve had people approach me to rent my horse to use them…that didn’t seem safe’*

(Participant 12)

##### Subtheme: Knowing the Horses

One standpoint that the vast majority of practitioners agreed on was the importance of really knowing horses, both as a species and as individuals. Practitioners acknowledged that a high degree of skill, which could not be acquired through simply completing ‘a course’, was required; this needed time to be developed. The degree of equine attunement was sometimes likened to a marriage, a skilled stockman, or that of a parent and child, where minute changes in behaviour were readily detected. The ability to ‘feel’ when something is not quite right allows practitioners to make precautionary pre-emptive changes that often prove to be well-founded. This heightened awareness is perceived as key for session safety for all and is acknowledged as a constant work in progress, as these practitioners demonstrate:

*‘…that day in day out knowing your animals…the same way, you know, farmers know their livestock is the same kind of principle, isn’t it? Just familiarity and it means that you pick up on things before they become issues…nothing is ever getting to the point where the horses are getting unhappy or uncomfortable’*

(Participant 12)

*‘you need to know your horses, you need to observe, and observe, and observe and then better observe, you know, when you think you know them, give yourself a kick and look again. It’s really important that, in terms of relationship, we notice them and they notice us’*

(Participant 13)

Reducing EAS to ‘simply adding a pony’ also diminishes the richness, professionalism, complexity, and added effort required to provide a quality human service, while at the same time caring for a large sentient being with their own complex needs. There is a sense among practitioners that those outside EAS may regard the field as unproven or of limited value beyond the feel-good factor or, as one practitioner described it, a *‘bit of a jolly*’ *(Participant 4).* This is exacerbated by the perceived less-than-solid evidence-base, as well as the lack of understanding as to why this may work. Many practitioners report that funders often need to ‘see it to believe it’ with those already familiar with EAS being the greatest supporters and advocates. In fact, some practitioners reported providing free taster days or introductory sessions as a successful way to engage funders or organisations.

#### 3.2.5. Theme 5. EAS as a Field Is Vulnerable

While practitioners are overwhelmingly in favour of incorporating horses into services, there is widespread recognition that challenges exist. The theme of vulnerability encapsulates the precarious position that many practitioners portray both in personal terms and for the field in general.

##### Subtheme: Sustainability

Probably the most pressing challenge that practitioners mentioned was making EAS a sustainable career or business. While there were issues with attracting clients to services, it was the costs associated with horses themselves that were a substantial cause for concern. In addition, the physical and outdoor nature of the work was considered to be very demanding, as this practitioner described:

*‘It’s hard work caring for horses and managing a practice. If you were going into therapy or counselling work or coaching work without horses you would be perhaps paying for a room, you know your therapy room, and you may push the hoover around a couple of times a week…when you are working with horses it’s 24/7, managing things that can have their own crisis, can be unwell, you are managing the elements and how your world is affected by the winds, rain storms’*

(Participant 15)

Practitioners often reported that their passion for the work pushes them to continue their service even when the monetary return is low, with one practitioner commenting:

*‘There are easier ways to make money’*

(Participant 14)

Having another job, or source of income besides EAS, was also not uncommonly reported upon. Many practitioners indicated that they provided some services at a reduced price, or even free, when costs to clients are prohibitive, particularly in cases where parents or clients are financially vulnerable or funding has ended. A reliance on the goodwill of volunteers was found to be common, with one practitioner reporting feelings of guilt at not being able to provide competent volunteers with some kind of remuneration. The all-consuming nature of providing EAS, along with a precarious financial situation, can be a problem long term, particularly for women who may have caring responsibilities at different life stages or those with health issues:

*‘I think even the longevity of it…there isn’t a long shelf life in this industry, and I think a lot of it has to do with the physical side. Even the amount of time it takes. It might work for people until they have a family and kids and then all of sudden it doesn’t’*

(Participant 8)

##### Subtheme: EAS as a Disjointed Field

Practitioners expressed very serious concerns about EAS as a field. While EAS at its best was considered to provide opportunities for connection, building relationships, and enhancing wellbeing, the field was often viewed by practitioners as disconnected and disorganised. Ambivalence in the field was seen as a systemic issue. However, the foundations of this ambivalence varied considerably. Practitioners spoke of some people ‘jumping on the bandwagon’, seeing EAS as an easy way to make money, or as a way to diversify their main equestrian business. Others were perceived as well-meaning and eager to help others, but were unaware of their ‘unconscious incompetence’. A minority of those entering the field may not even consider training necessary, one practitioner evidencing this point through what they describe as a typical Facebook quote *‘Do I have to do a course to do this, I’ve been working with horses and children all my life’* (Participant 1). These attitudes are regarded as damaging to the field. In addition, the lack of consensus regarding core competencies in EAS serves to exasperate these issues:

*‘Yeah I think there’s a kind of disconnect between competencies, like, there isn’t an established competency framework that is known out there in the rest of the world…so there’s something around standardising and competency frameworks [needed]’*

(Participant 15)

A few independent international organisations, such as IAHAIO and HETI, were perceived by the practitioners in this study positively, particularly in terms of raising awareness and providing educational material. However, their power to influence standards beyond best practice guidelines was considered to be limited. Some practitioners spoke of feeling isolated, of being on the fringes, or not knowing where to turn to for support. The lack of an independent governing body with expertise in EAS to help practitioners professionally was seen as problematic for a good number of practitioners, especially in terms of creating a professional profile:

*‘If there was a kind of one stop shop for professionals in this field, I think that would be really good. Now there could be, but I haven’t found it, you know, I still don’t know who to join…that’s really something that would be amazing to have, a professional network, professional development, when you join an organisation you know you’re really getting value’*

(Participant 2)

Many practitioners took solace in the knowledge that they had a good grounding in the underlying therapy or service provided, along with relevant equine and EAS qualifications. A number, however, found the lack of standards in training a huge worry in terms of being able to develop professionally:

*‘How do I benchmark myself, how do I actually know that I am actually being the best that I can be? There’s nothing. It’s like water. There’s nothing to mark against’*

(Participant 2)

Presently, there is no independent governing body within EAS that has the scope, expertise, or powers needed to set and maintain ethical or competency standards of practitioners specific to horse–human work. This, together with the variability in the field, has not gone unnoticed by many practitioners interviewed:

*‘And that comes back to this, sort of idea of consistency, I think. And not that it has to be the same [ training or service] but just, that it should be consistent. And I think there’s a lot of stuff out there that is, like you know, even distance learning courses that…there isn’t kind of consistency along peoples’ training or background…that’s a little problematic, and there only has to be, you know, a bad accident for insurance to be like, we’re just not doing that anymore and then we’re all going to be in a hole aren’t we’*

(Participant 12)

Practitioners were found to be very aware of the degree of variability in training in the field. Training to practice EAS can range from one to several days of in-person training, hybrid in-person/online courses, online-only courses, block release type courses (combining weekly online work with monthly in-person training over an extended period) up to intense traditional full/part-time diploma or postgraduate offerings. Many courses cost a considerable sum of money, and are intensively marketed, with little (independent) indication as to the quality or comprehensiveness of the training provided; this means many vulnerable learners resort to a ‘leap of faith’ type training decision. Most training providers, regardless of course length or content depth, advertise courses as ‘qualifying’ or preparing practitioners to practice. This ‘one-course’ mentality of providers is not reflected in the multiple trainings and CPD (continued professional development) that the practitioners here and elsewhere have reportedly undertaken. A caution of ‘buyer beware’ was implied by many. While some practitioners favoured specialist training in different aspects of EAS, others considered experiencing different practitioner methods as important. Regardless of the type of training undertaken, all practitioners noted real issues with the training depth, quality, and experiential content of many courses advertised:

*‘…fundamentally everyone can pretty much do their own thing. But I think we have to look carefully at….what’s the difference between a practitioner who’s done a 5-day online course and somebody who’s done a complete [...] training with practice clients and supervision and everything else. I’m afraid there will be a difference. Because there’ll be a difference in the quality of the knowledge, extent of the knowledge and extent of the training……because 90% of the learning is being with the horses’*

(Participant 3)

In addition, this practitioner highlights the stark differences between assessing competencies in EAS with other professions:

*‘[in other professions] you take some exams and then you have finally a face-to-face interview [with a panel] and then you qualify. What that gives everyone outside is, they know the standard of that qualification…and that’s what we don’t have. You know, we have all sorts everywhere’*

(Participant 3)

While the vast majority of participants in this study felt that having knowledge and experience of horses was crucial for the work, some practitioners questioned the type and relevance of equine education that some practitioners may bring to the table. A person’s ability to compete in equestrian sport, for example, was not regarded as proof of having adequate equine skills or knowledge. On the contrary, the objectification of horses in equestrian sport may conflict with the more relational position of EAS:

*‘There would be very few that I would have worked with that would be open to seeing horses in this role, in this dynamic way…Unfortunately in the equine industry, for a lot of people, horses would be robotic, they’re a commodity, you know, which is the bad side of the equestrian industry, probably why I left’*

(Participant 16)

There was a tacit assumption through the interviews that the ethos of EAS was quite different from the mainstream horse industry. An implied assumption of practitioners was that EAS should be viewed as a multispecies interpersonal space with beliefs, attitudes, and values aligned with good wellbeing for all, rather than as a cold ‘use’ of horses to help humans. Included in this worldview was the need to support practitioners’ work and wellbeing in various ways, which was seen as lacking in the field by many. Some practitioners did feel that they were receiving good support, most commonly through a network organised by, or connected to, a particular training programme or EAS organisation. An example of some of the benefits of such a network are described here:

*‘[Organisational CPD/Events] so you’re all the time getting self-care within it which is massive in this field, absolutely massive. So yes, there’s a self-care element of it, a wellbeing element of it. And for me, that’s so important. And the [organisational] network, I suppose we nearly have a community…and that for me is so important and we all back each other, it’s very supportive’*

(Participant 16)

In addition to peer support, many practitioners cited supervision as a possible way to assist practitioners going forward. Many professions, particularly those in mental health, require supervision to practice. Some practitioners spoke of how supervision allows practitioners to ask questions and discuss difficult cases to learn and grow from. With the absence of an umbrella governing body, supervision is seen by some as a way to develop strong competencies, provide support, and ensure some degree of quality control within EAS:

*‘I would like to see us as an industry developing a professional standard that, is like a supervision that you would have in other types of structured organisations, because it’s nice to have someone to run things by and it’s important for quality control. And I think a lot of people are working in isolation, which probably makes them quite vulnerable because we do get disclosures…so it’s quite difficult to navigate. I think I find it quite difficult to navigate’*

(Participant 12)

Ultimately, some degree of professionalisation of EAS is considered essential for the credibility of the field, to set standards, and to protect all involved. Some practitioners, already part of a profession, see clear advantages in having this additional designation, both in terms of public confidence and credibility when securing funding. Without the necessity to be ‘competent to practice,’ it was felt by some that the field will remain vulnerable to malpractice and reputational damage by association, a situation to which more than one practitioner has been witness. The ‘one bad experience could ruin it for everyone’ belief highlights the perceived vulnerability felt by practitioners.

## 4. Discussion

This study explored, for the first time, the lived experiences and perspectives of Equine-Assisted Services practitioners using a qualitative methodological approach. The research aimed to determine practitioners’ opinions regarding the horse–human bond, the possible mechanisms underlying the ability of horses to benefit humans, as well as identify the challenges facing the field of EAS more generally.

This study of 15 EAS practitioners yielded 5 common themes, named here as: 1. Developing strong and lasting connections through horses; 2. It’s all about the relationships; 3. Horses enrich the service; 4. EAS is more than just adding a pony; 5. EAS as a field is vulnerable. These themes were intertwined across interviews, despite the diversity of EAS and range of practitioner backgrounds (e.g., mental health, physical health, learning, therapeutic riding) presented here. This perhaps indicates the presence of an underlying ‘EAS culture’, distinct, yet with some underlying social norms and similarities to ‘horsey’ or ‘yard’ culture, as described by social science scientists [30,31,32]. Indeed, the horse may be the inducement to help this diverse community find common ground in an otherwise fragmented field. Conversely, a collective focus on wellbeing, including an acute awareness of the issues pertaining to both clients and horses, may underpin practitioners’ views and experiences. Regardless, the ‘horse effect’ as described here in its pure, as well as managed, form, appears to construct similarities in understanding and knowledge for practitioners.

### 4.1. Developing Strong and Lasting Connections Through Horses

The findings from this study give important insights into the role that horses play in building connections for clients and practitioners. Connection, or connectedness, as described and understood here, can be considered an active engagement with a person, animal, or environment, resulting in enhanced wellbeing [33,34]. In this study, this description of connection is also extended to include connection to oneself.

Conversations with participants regarding connection typically veered between the influences of horses on practitioners’ and clients’ connectedness to the self, others (including horses), the environment, and how this was realised. The relative ease with which horses seem to facilitate the types of connection described here is not new [13,35]. However, it has important implications for understanding the possible underlying mechanisms at play. E.O. Wilson’s (1984) biophilia hypothesis [36] proposes that humans have an inherent connection with nature, including animals, and that this connection is beneficial for wellbeing. In addition, the necessity for connections with others is considered innate [37], although proficiency in building connections can be developed [38]. At present, a large body of research supports the critical role of social, self, and nature connectedness, as well as a more general connection, for wellbeing (for review see [39]). It seems plausible that horses may play a role in facilitating this process.

Interestingly, a connection deficit phenomenon is currently being experienced in society [40], with symptoms of increased loneliness and social isolation [40,41], depression [42], internet addiction [43] and societal violence [44,45]. There is also a growing move towards more superficial or suboptimal, ‘pseudo-connections’ found within the social media space [46]. Our inherent inclination towards connectedness can additionally be thwarted by an array of barriers, e.g., economic, health, psychosocial [47,48,49,50,51]. For some individuals, motivation to connect with others can also be low, especially for those with poor health or wellbeing [52]. The findings presented here suggest that horses may be able to act as a potent ‘hook’ or focus, capable of counteracting some psychological barriers to connection. Horses therefore may serve to open clients up to the possibility of connection, an essential step that can be missed through other interventions.

Connection is not a fixed phenomenon. Connectedness in one area can spread to others. For example, nature connectedness has been shown to increase social connectedness [53]. EAS may have the potential to foster a rich general sense of connection on multiple fronts. A mindfulness-like state, as described by some practitioners in this study, has been linked elsewhere to greater connectedness, improved wellbeing, and even flourishing in healthy populations [54]. General connectedness, also regarded as the essence of more specific types of connectedness, has not only been posited to be protective for positive wellbeing [55], but also self-esteem [56] and emotional regulation [57], outcomes that align with many practitioners’ accounts. Based on these descriptions, opening the door to connectedness and subsequent connection could be considered as one of the great strengths of EAS and a possible mechanism of change that warrants further exploration.

### 4.2. It’s All About the Relationships

Although important in its own right, connection also makes a significant contribution to building relationships. Relationships, more substantial entities than simple connections, can be defined in their basic form as a series of enduring interactions between two or more individuals that can mutually influence the subjective experience of the other [58]. The practitioners in this study depicted a rich relational space, full of opportunities to experience and learn about what constitutes healthy relationships. The multiple parallel relationships described here may serve to create a relational immersion, somewhat akin to an intensive language course, where new knowledge and practice merge to heighten learning. However, immersion in the context of EAS may have a less intense quality, with practitioners speaking of working at the clients’ pace, talking less, and allowing clients ample time and space to grow. This points to learning within EAS as an organic process. Paradoxically, it is this time and space that appears to allow clients to quickly gain the confidence needed to reach out and establish relationships.

Allowing a level of control or autonomy, as illustrated here, has been shown to increase trust [59]. Trust building is an essential component in any healthcare strategy; uncertainty and vulnerability characterise many service user situations, with trust mitigating the negative effects of these influences [60], while also serving as a basis for building good relationships. Demonstrating trustability through observing a practitioner’s relationship with another being (social or inferential learning), has a two-fold benefit. It provides the client with information as to the trustworthiness of a person [61,62], while also exposing the client to positive ways of acting or relating to others [63,64].

Practitioners’ widely held view that ‘it’s all about the relationships’ is particularly salient given the strong links between relationships and wellbeing (for review see [65]) Relationship-building opportunities in an EAS context come with many benefits. Firstly, having a bond with a horse can act as a stepping stone for developing bonds with humans [66], in this respect serving as a ‘social lubricant’; the social facilitation effect is well documented in the human-canine literature [67] and encapsulated by psychologist Boris Levinson’s innovative inclusion of his dog, Jingles, within the therapeutic setting [68]. The findings of this study suggest that horses may be able to serve the same function. Secondly, the rapid development of a strong therapeutic alliance, as typically characterised by practitioners, is associated with positive outcomes across a range of interventions and is regarded as a strong predictor of success [69], pointing to the possibility of social support provided by horses as one of the possible mechanisms underlying this phenomenon. As a good therapeutic bond and social support are both associated with positive therapeutic outcomes [70], horses may have a dual role. Indeed, social support may provide a mechanism through which clients not only better cope with adversity, but can also grow or thrive [71], a feature of EAS touched on elsewhere [72].

Developing several multispecies relationships, as described by many practitioners, affords clients the chance to practice relational skills, considered key for generic skill success [73]. The benefits of this kind of experiential, and often embodied relational, learning, typical in EAS, are widely recognised [74]. Additionally, some relational skills, such as learning body language, can be more readily and effectively taught initially through indirect horse observation, eliminating the fear of judgement associated with more direct participation, whilst breaking learning down into easy-to-manage constituents [75]. Building a relationship with a horse may be easier to achieve than with humans due to the cleaner communication possible from body language alone. This gentler, relational education, which mimics real-life situations, makes the transference of skills and attitudes to everyday life a more seamless and viable prospect. Furthermore, learning about another being can help foster empathy [76], another important component of relational learning. As empathy towards humans and animals has positive associations, learning empathy through horses may promote greater overall empathy [77], yielding further benefits [78,79]. Other skills typically developed through EAS (e.g., maintaining safety and boundaries within the human and equine space, reciprocity, mutual regard, learning to relate and listen to the horses, and responding appropriately), are all important relational skills that are transferable and relevant to everyday life [80,81,82]. The strong relational underpinning of EAS lends itself to more client-centred, holistic ways of being and growing, allowing individuals to be seen and valued within a safe space [13], thereby fostering self-acceptance [83]. This provides an ideal blueprint for growing healthy relationships both within and outside EAS settings.

### 4.3. Horses Enrich the Process

The practitioners in this study spoke of a wide range of expected and unexpected ways in which horses enrich their services. Some practitioners stated that the same level of work could not be achieved without horses, suggesting that EAS is an efficient provision, both in terms of time and quality of progress. There was also a consensus that EAS can meet the needs of people in unexplained or indirect ways, including highlighting needs that clients or practitioners were not previously consciously aware of. How horses were able to achieve this, however, was considered to be obscure or shrouded in mist. Some practitioners believed that horses somehow simply knew how to react to people, almost like an innate ‘sixth sense’. Others regarded horses as catalysts for seeing things more clearly, thereby initiating change, a finding touched on in the literature [84]. Either way, there was an assumption that something very real, but unexplainable, happened during human–horse interactions. The mention of ‘magic’ occurred sporadically, perhaps in an attempt to explain the unexplainable, rather than give credence to an ‘otherworldly’ phenomenon. Being able to ‘see it all in front of you’ embodies these eye-opening moments experienced in EAS.

One of the most striking patterns observed through the data was the strong motivational effects observed. This effect was not just characteristic of the initial horse–human interactions phase, but one that continued week after week. High motivation and commitment in clients have been reported in other EAS studies (for a scoping review see [66]); this is not a typical feature of other health and wellbeing interventions, however [85] from the stories gathered in this study, connection and relationship development provide a strong social support effect that nurtures motivation to engage, a link already verified in the non-EAS literature [86]. However, from the findings presented here, motivation may also be a continuous, enduring feature of EAS, starting even before a tangible connection has been developed. It could be that the bond, or potential to bond, with a horse acts to sustain motivation where it may otherwise have waned. Related to this, social support, motivation to engage, and wellbeing are enhanced by improved self-esteem and self-efficacy [86], a common co-benefit of equine interactions reported both here and more widely [87,88,89]. The fact that progress in EAS is not often linear was frequently implied. Although initially counterintuitive, the types of uncertainties and unexpected outcomes experienced in this field are characteristic of complex systems [90], the criteria for which (non-linear and unexpected outcomes, interconnectedness, many moving parts) EAS naturally meets. For practitioners in this investigation, horses appear to serve as the incentive to keep going when things get tough, promoting perseverance through sustained challenges; this suggests that EAS may also play a role in building resilience. All of these elements working in tandem appear to serve as a complex system capable of providing strong growth and wellbeing benefits to clients. This growth, however, cannot occur without the strong relational underpinning of EAS already discussed. As mentioned earlier, research is clear on the benefits of quality social support [91], including that acquired from animals [92,93]. The inclusion of equids as social support, with the advantages of being non-verbal and less taxing than humans to interact with, may provide a uniquely motivational environment [35].

Practitioners frequently spoke of the positive mood and calming effect of horses on clients. These effects have also been reported in a number of qualitative studies. Within the human–animal bond literature, there has long been an established link between human health and pet ownership, with positive effects, particularly with dogs, on cortisol levels, blood pressure, oxytocin, heart rate, and cardiovascular health (for review see [94]). These, along with frequently cited social influences [67], position dogs as beneficial within a biopsychosocial context [94]. The findings here and elsewhere point to the possibility of similar effects from horses. In addition, having another being to focus on in a normally dyadic situation, such as a therapy session, could provide added benefits. It may be that joint attention (in this case, sharing a horse experience with another), along with the less intense three-way nature of horse–human interaction, can reduce some of the pressure commonly associated with more typical dyadic therapeutic relationships, promoting a sense of calm. It seems likely, therefore, that horses can enhance services through their mere presence [92,95,96], especially through transitions or challenges within sessions. Again, these benefits have been noted in other species, including dogs [97].

Horses may also enrich the EAS process by providing numerous opportunities for safe touch, e.g., riding, grooming, stroking. Touch has important implications for health. For example, kangaroo care, the prolonged skin-to-skin contact promoted for premature infants, is associated with improved health and autonomic regulation outcomes [98]. Touch has long been considered important for child development and social cohesion, as well as for various physiological processes [99,100,101]. Research in this area has not been restricted solely to human touch. Studies have found that petting a dog for a short period can have an immediate positive effect on stress levels and mood [102]. It seems logical that touching a horse through petting or the more embodied experience of riding could also have a positive influence on stress or affective state via oxytocin production or biopsychosocial effects [103], inadvertently fostering social cohesion and prosocial behaviour in the process [104].

A key feature of the first 3 themes identified in this study (connectedness, relationship, enriching benefits received through EAS), aligns well with an overarching emotional intelligence development as a possible underlying mechanism within EAS [105]. Emotional Intelligence (EI) has been described as the ‘ability to monitor one’s own and other people’s emotions, to discriminate between different emotions and label them appropriately, and to use emotional information to guide thinking and behaviour’ [106]. According to Salovey & Mayer [106], emotional intelligence, through the lens of cognitive ability, comprises awareness of own emotions, management of own emotions, motivation of oneself, awareness of the emotional state of others, and successful navigation of relationships. Hughes et al. [107] later updated this construct to include set behaviours or strategies associated with successful emotional regulation. It seems that EAS may have an inherent, albeit incidental, affinity towards the building of emotional intelligence. Many of the skills and competencies needed for EI (observation and listening skills, self-awareness, emotional awareness and regulation, relationship building, and modelling) are intrinsic to EAS. This indicates a potential mechanism of change not commonly discussed in the literature. EI’s links to subjective wellbeing [108], mood [109], and self-esteem [110] also align well with many practitioners’ experiences and perspectives. This finding is significant as poor mental health is associated with lower emotional intelligence [111], impeding a person’s ability to form healthy or positive relationships. EAS has, therefore, the potential to reverse this trajectory, improving wellbeing in the process. Although a small number of EAS studies have delved into EI [14,112], there may be significant merit in exploring further the synergy that may exist between the two, including possible ways in which this may be enriched through careful inclusion of EI in programme development. The incorporation of strategic and deliberate EI approaches into existing EAS models through enhanced EI training of practitioners may provide one robust strategy, among others, for developing greater efficacy within services and the wellbeing of clients going forward.

### 4.4. More than Just ‘Adding a Pony’

The fourth theme of this study explored EAS as ‘more than just adding a pony’. The incorporation of a horse into services was viewed by practitioners as highly complex, and there was the inference that this complexity was not adequately appreciated by others. Practitioners frequently spoke about EAS as requiring substantial skill sets that demand competencies traversing horse, client, service, and environment. Understanding the dynamic nature of EAS is perceived as crucial. This view is not without basis. Activities that include riding, or simply interacting with, horses are not without risk [113,114]. An understanding of equine welfare and behaviour, particularly an ability to recognise heightened affective states, significantly impacts risk within normal equestrian activities [115]. If you add to this the additional skillsets needed to rehabilitate and manage the significant number of rescued, retired, or traumatised horses often employed in EAS [16], the importance of deep-rooted equine and EAS-related knowledge becomes clear. Understanding horses, as well as the intricacies inherent in horse–human interactions, was seen as a way of protecting equine welfare and a route to providing a safe service. Competencies discussed by practitioners were not restricted to those related to horses. There was a concern that some practitioners might have a casual attitude towards a potential clientele group, with a ‘who would I like to work with’ as opposed to a more professional ‘who am I most qualified to work with’ stance. In this sense, the ‘more than just adding a pony’ was concerned with skills that may be overlooked when working with vulnerable clients. Part of this concern may stem from the growing, yet long-standing, provision of riding for the disabled within many riding establishments. Although the official Riding for the Disabled Association (RDA) has well-established coaching pathways, procedures, and practices in place [116], a plethora of unofficial ‘add-on’ riding for people with various disabilities has mushroomed in recent times. This, together with some EAS provisions, both mounted and ground-based, operating with little or modest specialised training [16], lends support for this concern. A lack of knowledge of contraindications to mounted work for those with physical issues [117], or conversely, poor mental health awareness [118], were cited as issues associated with limited formal training, possibly fostered by the naïve attitude of just ‘adding a pony’. Practitioners also spoke of knowledge deficiencies regarding client groups, services, and general competencies to practice. These concerns pose serious questions for clients’ physical and emotional safety and raise ethical concerns about exposing vulnerable populations to unnecessary risks associated with suboptimal expertise. From these findings, raising awareness of EAS as a specialist endeavour requiring a unique set of skills in both the equine and human realms is essential if EAS is to be viewed, internally and externally, as more than just adding a pony.

### 4.5. EAS as a Field Is Vulnerable

Despite practitioners’ compelling stories of success and dedication to their work, a clear undercurrent of vulnerability regarding individual practitioners’ situations, as well as the greater field of EAS, emerged from the data. Whilst the perceived benefits are great, these were matched by the concerns.

Probably the most pressing issue faced by practitioners is financial and labour sustainability. Including horses in any service is costly, both in monetary and labour terms [119], and this must be reflected in the price of the service. However, finding clients capable of paying the market value for such services can be challenging. These challenges are somewhat lessened, but not eradicated, for professionals (e.g., occupational therapists, mental health professionals, educators) who incorporate horses into their work. While a small number of practitioners reported some confidence regarding their financial future, the consensus was that of a precarious situation, to some extent dependent on funding or fundraising, Similar findings have been reported elsewhere [120]. Even for those with more stable incomes, the inner pressure to provide discounted services to those who were financially vulnerable themselves was great. Employed practitioners were not exempt from this vulnerability; well-paid employment was deemed hard to come by. From these accounts, practitioners may be viewed as practicing a degree of self-inflicted exploitation, similar to that reported by marginal, traditionally run family farms [121]. This self-exploitation can manifest as an intense motivation to provide service despite long hours, poor monetary return, or even taking a second job to get by financially. Indeed, a deep motivation to work with horses in the wider equine industry has likely contributed to a culture of low pay and even employee exploitation [122,123,124,125]. A sense of financial vulnerability also appears to extend beyond the service itself. Practitioners spoke of the universally high cost of education within EAS, despite its variability and lack of a governing body to ensure standards. This can leave practitioners open to the risk of financial and educational exploitation through expensive and potentially substandard training. Obtaining additional training to fill in the gaps places a further financial burden on practitioners already under pressure. While working for low returns for the greater good is an admirable endeavour, it may not be sustainable in the long run. As one participant stated: *‘There are easier ways to make money’*; this may well capture the financial conundrum many practitioners face.

The vulnerability of EAS also appears to stretch to the area of standards. An uneasiness regarding health and safety risks, competency to practice, and lack of regulation across the EAS field have been raised by others [112,126], as well as within the wider animal assisted services field [118,127,128,129], leading to calls for greater professionalisation in all animal-assisted work [130]. With no governing body to oversee the training or provision of EAS, the standards and quality of service within the sector, even among accredited courses and programmes, are difficult to determine. In addition, a lack of standards allows unscrupulous or unqualified practitioners to provide services for which they lack the competencies to carry out safely. This leaves clients open to unnecessary risks, increasing the likelihood of harm and potentially causing reputational damage to EAS. These risks are not unfounded, as withdrawal of funding [118] and insurance issues [131] have both been attributed to poor EAS practices. Although several registers (e.g., Human Equine Interaction register (HEIR), Athena, Horses in Education and Therapy International (HETI)) have been set up to try and address the issue of standards, without a governing body, a consensus regarding a competency framework and a level of regulation, these registers can only offer limited assurances regarding practitioners’ fitness to practice. Until the area of standards has been adequately addressed, EAS will continue to be vulnerable to the reputational damage alluded to here, should adverse incidents occur.

Practitioners’ perceptions of support within EAS and the service provided were also a cause for concern. Participants described a somewhat disjointed and disconnected field, lacking the foundation of common ground and a unified voice. Although some practitioners felt supported, this backing was often within a training organisation to which the practitioner belonged. Exceptions to this, such as HETI, providing free educational webinars to members as part of its EAS promotion and development strategy, have the potential to fill this void. Growing practitioner awareness of this support, as well as developing further organisational support strategies, may be the key to addressing some practitioners’ feelings of isolation and building greater connectedness and trust.

Concerns were also expressed regarding animal welfare and public perceptions. Although practitioners view animal welfare as being extremely important [16], with horse wellbeing a key part of providing a congruent service for clients, many implied that this ethos was not consistent across providers, leaving the field open to challenges regarding social licence to operate (SLO). SLO within equestrianism refers to the public level of approval for equine and equestrian activities, allowing such industries the freedom to self-regulate without outside interference [132]. Although SLO in this sense has mostly been concerned with competition and leisure riding, any activity involving horses, including EAS, may find itself under scrutiny. Equestrianism is currently experiencing strong challenges to its SLO. Media scrutiny in horse sport and racing has uncovered draconian training practices, compromised welfare through management or medication, failure to enforce organisational standards, and lack of accountability in terms of equine wastage and inadequate post-career aftercare, amongst other issues [133,134,135,136]. In contrast, most of the EAS practitioners’ values, attitudes, and beliefs articulated here were not aligned with those of the mainstream horse industry; aversive practices were viewed as counterproductive, and the objectification of horses was not perceived as aligning with a wellbeing for all stance. These are positive findings in terms of EAS, indicating a shift away from some aspects of mainstream horse culture towards wellbeing-centred practice. However, much equine training and handling remains centred on the values, attitudes, and beliefs inherent in horse sports, which hold a goal or competition, rather than a wellbeing focus. As such, EAS is in danger of being tarred with the same brush through association. In light of these threats, it is clear that EAS must endeavour to forge its own identity, including educational specifications (both equine and service) and operating standards, coherent with a wellbeing stance.

### 4.6. Future Recommendations

From these findings, it is clear that addressing standards via an evidence-based competency framework is an urgent priority. Ideally, this framework would be developed through consensus by consulting a wide range of experts in EAS, as well as those in adjacent fields, including education, health and wellbeing. A transdisciplinary approach to standards would help fill in some of the gaps in knowledge alluded to in this study. Additionally, a governing body, with powers to regulate and enforce standards, could provide EAS with the level of professionalism needed to inject confidence into the sector. This could also provide practitioners, as well as clients and funders, with a reference point regarding the standards of practice and ethics expected. The resulting professionalisation would enable EAS to be better recognised by government departments and other organisations, for funding purposes, facilitating more consistent funding streams. Recognised standards would also empower researchers to create studies that reflect an accepted best practice, allowing results to be more generalisable to typical clinical settings. Conversely, research could better inform practice more readily in such circumstances. An independent governing body would also enable the field to come together with one voice when tackling difficult issues. The findings here suggest that horses play a role in connection and EI development. Further research could delve deeper into these possible mechanisms of change to create targeted strategies capable of taking advantage of these natural tendencies within EAS.

### 4.7. Limitations

This study has several limitations that should be acknowledged. Firstly, the practitioners included in this investigation came from the UK/Ireland and may not characterise a more international experience. Secondly, practitioners here were experienced in working with horses and also had specialist training in the service that they were offering. The majority had extensive EAS training beyond a single course and had indicated, explicitly and implicitly, a commitment to CPD or lifelong learning. The vast majority did not provide another equestrian service beyond EAS. Although these characteristics provide a good basis for attaining rich accounts of working within EAS, the voice of those starting out in the field, or working from a more traditional equestrian ethos (i.e., as part of a riding school provision), may be lacking. Most of the recruitment was self-selection; participants indicated an interest in taking part based on participation in a previous study, contact with the researcher, or through social media advertisement. This may have created a bias in terms of practitioners interested in taking part in research, or those who frequented social media platforms that promoted EAS or EAS research. This may have excluded practitioners who did not have an online presence or who worked more in isolation. Finally, the findings of this diverse sample may have left out some of the subtleties associated with the different categories of services. Further research could delve further into the differences, as well as similarities, associated with the distinct EAS groups.

## 5. Conclusions

Practitioners are uniquely positioned to provide a valuable insight into the field of EAS due to their close engagement with clients, horses, and outcomes observed. This study highlights key relational, social, motivational, and supportive features of incorporating horses into services, bringing us closer to identifying some of the underlying mechanisms possibly at play in helping to improve human wellbeing. However, the findings also depict a complex horse–client–practitioner dynamic that requires competencies in both human and horse domains to ensure the wellbeing and safety for all. In addition, practitioners have considerable concerns regarding the EAS field, particularly with respect to service and professional integrity, training, and standards. Future research must take into account these challenges, as well as continue to explore factors that influence efficacy of EAS if the field is to move forward as a valid, evidence-based wellbeing strategy.

## Figures and Tables

**Table 1:** Practitioner Background.

Practitioner Background	*n*	%
*Geographic region*		
Northern Ireland	2	13.3
Republic of Ireland	6	40.0
England	7	46.7
*Age (in years)*		
30–39	2	13.3
40–49	4	26.7
50+	9	60.0
*Highest level of completed education*		
Secondary level	1	6.7
Tertiary level	2	13.3
Professional qualification	4	26.7
Postgraduate	8	53.3
EAS category (multiple categories) *		
Equine Assisted Physical Health	2	13.3
Equine-Assisted Mental Health	7	46.7
Equine Assisted Learning	13	86.7
Therapeutic/adaptive riding	5	33.3

* EAS category percentage is from the total number of practitioners (*n* = 15).

**Table 2:** Final themes, subthemes, and definitions.

Theme	Subtheme	Definition
**Developing strong and lasting connections through horses**	Connection to self through horses Horses facilitate connection to others	Horses act as catalysts for connection on many levels, from the initial spark felt between the client and the horse to improved connection to the self, practitioner, other service personnel, and even the equine and natural environment. The connections act as a web to situate or ground the client. This web of connection helps clients to more quickly feel at home, fostering feelings of safety, acceptance, and being a part of something more than themselves.
**It’s all about the relationships**	Building a strong therapeutic alliance Multiple opportunities to practice relationship skills experientially	A multifaceted relational space. EAS is all about relationships, from the horse-client relationship, practitioner-client relationship, practitioner-horse relationships as well as the multiple multispecies relationships that exist within the team. EAS provides multiple opportunities for practitioners to model good relationships as well as for the client to practice building their multiple parallel relationships that can be transferred to ordinary life.
**Horses enrich the service**	Horses and the EAS environment as unique motivators Dynamic experiential environment promotes growth	Regardless of the service provided, horses contribute something extra into the mix which results in a higher level of outcome that could be achieved via a non-horse service. How this is achieved can be hard to explain and is multifaceted, depending on the service, client, and/or practitioner, but most importantly the presence of the horse in the equation.
**EAS is more than just adding a pony**	Awareness needed of the strong and complex skillset requirement Knowing the client Knowing the horse	EAS is a complex system that requires specialist skills. All EASs consist of numerous ‘moving parts’ and uncertainties that need to be constantly monitored. This dynamic environment can provide added benefits but also increased risk which must be mitigated against.
**EAS as a field is vulnerable**	Sustainability A disjointed field	The general lack of clarity and cohesion along with some confusion within and across the field of EAS leaves the sector open to internal and external threats. This is further aggravated by issues of financial sustainability or vulnerability which is often cited or implied as a challenge in EAS.

## Data Availability

Data are contained within the article.
